# INTERP: Interpreter requirements needed for tissue plasminogen activator evaluations and resulting performance: a retrospective review

**DOI:** 10.1186/s42466-024-00319-2

**Published:** 2024-06-13

**Authors:** Julia Ting Bu, Dawn M. Meyer, Benjamin Shifflett, Brett C. Meyer

**Affiliations:** Comprehensive Stroke Center, University of California San Diego, 9444 Medical Center Drive, La Jolla, CA 92037-0979 USA

**Keywords:** Interpreter, Language, Barriers to access, Health disparities

## Abstract

**Aim:**

To examine the influence of interpreter service needs (IS) on rt-PA administration time metrics.

**Methods:**

Retrospectively reviewed prospectively collected data from Comprehensive Stroke Center database (January 2011- April 1, 2021) and EMR. Inclusion: Subjects for whom a “stroke code” was activated. Excluded in-house strokes. Baseline characteristics, frequency of rt-PA, rt-PA exclusions and time metrics, NIHSS were compared between patients who did or did not require IS. Analyses utilized ANOVA, t-Test, Brown-Mood Median Test, or Pearson’s Chi-squared test as appropriate.

**Results:**

Of 2,191 patients with stroke code activations, 81 had a documented need for IS. Rt-PA was administered in 9 IS and 358 non-IS patients. Median baseline NIHSS was higher in rt-PA group (9±8 vs 3±9, p<0.005). In IS patients, there were no differences in baseline characteristics between those who received rt-PA and those who did not, including median score for NIHSS aphasia (0±1 vs 0±1, p = 0.46). There were no rt-PA rate differences between those that did not and did require IS (17% vs 11%, p = 0.22). In patients with final diagnosis acute ischemic stroke, patients excluded from rt-PA solely due to being out of the window were more likely to have required IS (59% vs 35%, p = 0.003). Time metrics of rt-PA administration were not different in IS patients.

**Conclusions:**

There was no significant difference in frequency or time metrics of rt-PA administration in patients requiring interpreter services during an acute stroke code. AIS patients requiring an interpreter were more likely to be excluded from rt-PA on the basis of time.

**Supplementary Information:**

The online version contains supplementary material available at 10.1186/s42466-024-00319-2.

## Introduction

In an acute stroke, “Time is brain”. Language barriers can inhibit efficient emergency response. In our experience, acute ischemic stroke (AIS) patients requiring interpreter services during an acute stroke code can experience care delays in obtaining a qualified medical interpreter.

Current literature is nebulous regarding the effect of language interpretation service needs (IS) on acute stroke response and outcomes. Though rt-PA administration rate does not appear to be different [1] in patients requiring IS versus not, patients in the former group were more likely to be discharged to facilities [2] or discharged with more severe neurologic deficits [3] with worse quality of life indicators on follow up [4]. Another study found no significant difference in quality benchmarks and outcomes [5].

The purpose of this study was to examine the influence of requiring IS on time metrics of rt-PA administration among acute stroke patients found in one comprehensive stroke center database.

## Methods

We retrospectively reviewed prospectively collected data from our Comprehensive Stroke Center database (1/2011-4/1/2021) and EMR. The status of requiring interpreter services was identified by patient or healthcare proxy. Subjects in one of two primary hospitals for whom a “stroke code” was activated were included; in-house strokes and stroke code activations from satellite facilities were excluded. Baseline characteristics, frequency of rt-PA, rt-PA exclusions, and rt-PA time metrics were compared between patients who did or did not require IS. Analyses utilized ANOVA, t-Test, Brown-Mood Median test, or Pearson’s Chi-squared test as appropriate.

## Results

**Fig. 1:**
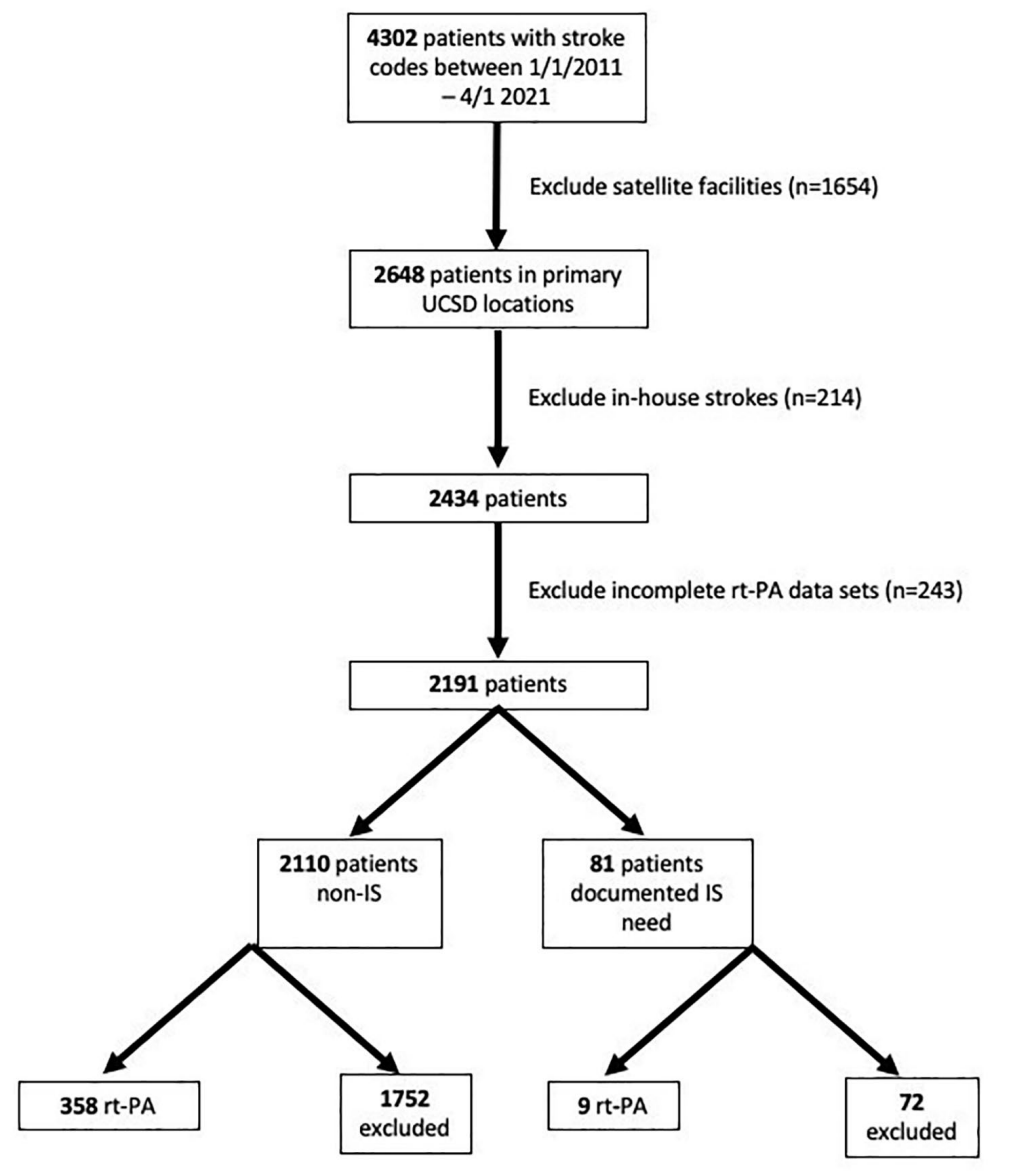
Flowchart of patient inclusion and exclusion criteria

There were a total of 4,302 patients with stroke codes between 1/1/2011 and 4/1/2021 (Fig. 1). Patients from satellite facilities (*n* = 1,654), who were in-house stroke codes (*n* = 214), or with incomplete rt-PA datasets (*n* = 237) were excluded. Of 2,191 patients remaining, 81 had a documented need for IS. rt-PA was administered in 9 IS and 358 non-IS patients.

Baseline characteristics of IS patients are shown in supplemental Table (1) In IS patients, there were no differences in baseline characteristics between those who received rt-PA and those who did not including median score for NIHSS aphasia (0 ± 1 vs. 0 ± 1, *p* = 0.46). Baseline characteristics of subjects who received rt-PA are shown in supplemental Table (2) Median baseline NIHSS was higher in rt-PA group (9 ± 8 vs. 3 ± 9, *p* < 0.005). In patients who received rt-PA, there was a larger amount of Hispanic ethnicity (*p* < 0.01) and hyperlipidemia (*p* = 0.04) in patients requiring IS.

There were no rt-PA rate differences between those that did not and did require IS (17% vs. 11%, *p* = 0.22). In patients with final diagnosis acute ischemic stroke, patients excluded from rt-PA solely due to being out of the window were more likely to have required IS (59% vs. 35%, *p* = 0.003). Time metrics of rt-PA administration were not different in IS patients (Table 1).

**Table 1:** rt-PA + Subset Analysis: time metrics by interpreter needs

	Interpreter Status	Number of subjects	Median time (minutes)	*P*-Value
**Onset to Arrival**	IS (-)	334	63.0	
	IS (+)	8	76.0	
	All	342	63.5	*P* = 0.65
**Arrival to Decision**	IS (-)	311	33.0	
	IS (+)	8	42.5	
	All	319	34.0	*P* = 0.45
**Arrival to Treatment**	IS (-)	334	53.0	
	IS (+)	8	75.5	
	All	342	53.5	*P* = 0.48
**Onset to Treatment**	IS (-)	334	128.0	
	IS (+)	8	169.0	
	All	342	128.5	*P* = 0.46

## Conclusions

Though this study found no significant difference in frequency or time metrics of rt-PA administration in patients requiring interpreter services during an acute stroke code, it adds to prior literature in showing that if patients required an interpreter, they were more likely to be solely excluded from rt-PA on the basis of time. Prior literature has shown disparities in discharge dispositions and outcomes [2–4]– our study suggests there may be disparities in how rapidly IS patients are being evaluated. Since rt-PA time metrics are not significantly different, one theory is that for patients with clear stroke syndromes, rt-PA may be offered under emergency consent. However, for mild strokes where further history is needed to elucidate degree of disability for rt-PA eligibility, requiring translator services may be a barrier to time-sensitive treatment.

One limitation of this study is the small single-center sample population. Based on our data, there was no significant difference in mRS between IS requiring patients who received or did not receive rt-PA. There was also a significantly larger amount of Hispanic ethnicity and HLD in patients requiring IS who received rt-PA. The clinical significance of these findings is unclear, but likely attributed to random variance in a small patient population where 30.3% of the community population is Hispanic [6]; of all patients in this time frame documented in need of interpreter, 77% required a Spanish interpreter.

We nevertheless add this study to the growing body of research on the impact that language has in a time sensitive field such as acute stroke care. Should our finding be replicated in other institutions or larger populations, this serves as a call to action for health care institutions to invest in language interpretation services. More work is planned in larger data sets to ensure resource availability and health equity to patients in need of interpreter services.

### Electronic supplementary material

Below is the link to the electronic supplementary material.

Supplementary Material 1

## Data Availability

The datasets analyzed during the current study are available from the corresponding author on reasonable request.

## Abbreviations

rt-PA: Recombinant tissue plasminogen activator
IS: Interpreter Service
AIS: Acute ischemic stroke
EMR: Electronic medical record
CAD: Coronary artery disease
DM: Diabetes mellitus
HTN: Hypertension
HLD: Hyperlipedemia
Afib: Atrial fibrillation
MRS: Modified rankin score
