# Supplementary material for: INTERP: Interpreter requirements needed for tissue plasminogen activator evaluations and resulting performance: a retrospective review
Source: Neurol Res Pract. 2024 Jun 13;6:31. doi: 10.1186/s42466-024-00319-2 (PMC11170910; doi:10.1186/s42466-024-00319-2)
Supplement: Supplementary file 1 — Supplementary Material 1 [file 42466_2024_319_MOESM1_ESM.docx]

|  |  | **TPA -** | **TPA+** | **Total** | **P-values** |
| --- | --- | --- | --- | --- | --- |
| **Age (years)** | **Median** | 73.5 | 68.0 | 72.0 | p = 0.7 |
| **Blood Pressure**  **(mmHg)** | **Systolic Median** | 142.0 | 153.0 | 142.0 | p = 0.76 |
|  | **Diastolic Median** | 78.5 | 82.0 | 79.0 | p = 0.44 |
| **Gender** | **Female** | 42 | 4 | 46 |  |
|  | **Male** | 30 | 5 | 35 |  |
|  | **All** | 72 | 9 | 81 | p = 0.66 |
| **Race** | **White** | 47 | 6 | 53 |  |
|  | **Asian** | 18 | 1 | 19 |  |
|  | **Black** | 1 | 0 | 1 |  |
|  | **Pacific Islander** | 1 | 0 | 1 |  |
|  | **All** | 67 | 7 | 74 | p= 0.85 |
| **Ethnicity** | **Not Hispanic/Latino** | 26 | 1 | 27 |  |
|  | **Hispanic/Latino** | 44 | 8 | 52 |  |
|  | **Unknown** | 2 | 0 | 2 |  |
|  | **All** | 72 | 9 | 81 | p= 0.26 |
| **CAD** | **No** | 60 | 9 | 69 |  |
|  | **Unknown** | 1 | 0 | 1 |  |
|  | **Yes** | 11 | 0 | 11 |  |
|  | **All** | 72 | 9 | 81 | p= 0.41 |
| **DM** | **No** | 43 | 5 | 48 |  |
|  | **Unknown** | 1 | 0 | 1 |  |
|  | **Yes** | 28 | 4 | 32 |  |
|  | **All** | 72 | 9 | 81 | p = 0.90 |
| **HTN** | **No** | 17 | 2 | 19 |  |
|  | **Unknown** | 1 | 0 | 1 |  |
|  | **Yes** | 54 | 7 | 61 |  |
|  | **All** | 72 | 9 | 81 | p=0.93 |
| **Hx HLD** | **No** | 39 | 6 | 45 |  |
|  | **Unknown** | 1 | 1 | 2 |  |
|  | **Yes** | 32 | 2 | 34 |  |
|  | **All** | 72 | 9 | 81 | p=0.12 |
| **Afib** | **No** | 57 | 8 | 65 |  |
|  | **Unknown** | 1 | 0 | 1 |  |
|  | **Yes** | 14 | 1 | 15 |  |
|  | **All** | 72 | 9 | 81 | p =0.77 |
| **Hx Stroke/TIA** | **No** | 39 | 7 | 46 |  |
|  | **Unknown** | 1 | 0 | 1 |  |
|  | **Yes** | 32 | 2 | 34 |  |
|  | **All** | 72 | 9 | 81 | p = 0.40 |
| **Current smoker** | **No** | 67 | 8 | 75 |  |
|  | **Yes** | 5 | 1 | 6 |  |
|  | **All** | 72 | 9 | 81 | p = 1.0 |
| **Current Alcohol** | **No** | 68 | 8 | 76 |  |
|  | **Yes** | 4 | 1 | 5 |  |
|  | **All** | 72 | 9 | 81 | p = 1.0 |
| **mRS 90 days** | **0** | 7 | 0 | 7 |  |
|  | **1** | 4 | 2 | 6 |  |
|  | **3** | 8 | 2 | 10 |  |
|  | **4** | 3 | 0 | 3 |  |
|  | **5** | 1 | 0 | 1 |  |
|  | **All** | 23 | 4 | 27 | p = 0.44 |
| **Aphasia (NIHSS score)** | **0** | 49 | 5 | 54 |  |
|  | **1** | 7 | 0 | 7 |  |
|  | **2** | 10 | 2 | 12 |  |
|  | **3** | 6 | 2 | 8 |  |
|  | **All** | 72 | 9 | 81 | p = 0.39 |

**Supplemental Table 1: Baseline Characteristics of Interpreter (+) Subjects by rt-PA**

|  |  | **Interpreter -** | **Interpreter+** | **Total** | **P-values** |
| --- | --- | --- | --- | --- | --- |
| **Age (years)** | **Median** | 68 | 68 | 68 | p=0.92 |
| **Blood Pressure (mmHg)** | **Systolic Median** | 153 | 153 | 153 | p =0.86 |
|  | **Diastolic Median** | 84 | 82 | 83.5 | p=0.99 |
| **Gender** | **Female** | 142 | 4 | 146 |  |
|  | **Male** | 216 | 5 | 221 |  |
|  | **All** | 358 | 9 | 367 | p=1.00 |
| **Race** | **White** | 280 | 6 | 286 |  |
|  | **Asian** | 20 | 1 | 21 |  |
|  | **Black** | 37 | 0 | 37 |  |
|  | **Pacific Islander** | 3 | 0 | 3 |  |
|  | **All** | 340 | 7 | 347 | p=0.65 |
| **Ethnicity** | **Not Hispanic/Latino** | 269 | 1 | 270 |  |
|  | **Hispanic/Latino** | 82 | 8 | 90 |  |
|  | **Unknown** | 5 | 0 | 5 |  |
|  | **Not reported** | 2 | 0 | 2 |  |
|  | **All** | 358 | 9 | 367 | **p=0.00012** |
| **CAD** | **No** | 298 | 9 | 307 |  |
|  | **Yes** | 60 | 0 | 60 |  |
|  | **All** | 358 | 9 | 367 | p=0.38 |
| **DM** | **No** | 284 | 5 | 289 |  |
|  | **Unknown** | 2 | 0 | 2 |  |
|  | **Yes** | 72 | 4 | 76 |  |
|  | **All** | 358 | 9 | 367 | p=0.20 |
| **HTN** | **No** | 153 | 2 | 155 |  |
|  | **Unknown** | 1 | 0 | 1 |  |
|  | **Yes** | 204 | 7 | 211 |  |
|  | **All** | 358 | 9 | 367 | p=0.46 |
| **Hx HLD** | **No** | 255 | 6 | 261 |  |
|  | **Unknown** | 4 | 1 | 5 |  |
|  | **Yes** | 99 | 2 | 101 |  |
|  | **All** | 358 | 9 | 367 | **p=0.04** |
| **Afib** | **No** | 271 | 8 | 279 |  |
|  | **Unknown** | 2 | 0 | 2 |  |
|  | **Yes** | 85 | 1 | 86 |  |
|  | **All** | 358 | 9 | 367 | p=0.65 |
| **Hx Stroke/TIA** | **No** | 277 | 7 | 284 |  |
|  | **Yes** | 81 | 2 | 83 |  |
|  | **All** | 358 | 9 | 367 | p=1.00 |
| **Current smoker** | **No** | 308 | 8 | 316 |  |
|  | **Yes** | 50 | 1 | 51 |  |
|  | **All** | 358 | 9 | 367 | p=1.00 |
| **Current Alcohol** | **No** | 299 | 8 | 307 |  |
|  | **Yes** | 59 | 1 | 60 |  |
|  | **All** | 358 | 9 | 367 | p=1.00 |
| **mRS 90 days** | **0** | 32 | 0 | 32 | p=0.34 |
|  | **1** | 41 | 2 | 43 |  |
|  | **2** | 28 | 0 | 28 |  |
|  | **3** | 22 | 2 | 24 |  |
|  | **4** | 17 | 0 | 17 |  |
|  | **5** | 6 | 0 | 6 |  |
|  | **6** | 17 | 0 | 17 |  |
|  | **All** | 163 | 4 | 167 |  |
| **Aphasia** | **0** | 165 | 5 | 170 |  |
|  | **1** | 74 | 0 | 74 |  |
|  | **2** | 50 | 2 | 52 |  |
|  | **3** | 69 | 2 | 71 |  |
|  | **All** | 358 | 9 | 367 | p=0.48 |

**Supplemental Table 2: Baseline Characteristics of Subjects who Received rt-PA by Interpreter Status**
